# Functional Outcomes of Radial Head Fractures Treated With Open Reduction and Internal Fixation (ORIF)

**DOI:** 10.7759/cureus.74801

**Published:** 2024-11-29

**Authors:** Muhammad Mannan, Muhammad A Hamid, Nayan Shrivastava, Rizwan Akbar, Abdul Rehman Sarwar

**Affiliations:** 1 Trauma and Orthopedics, Ghurki Trust and Teaching Hospital, Lahore, PAK; 2 Trauma and Orthopedics, Sheikh Zayed Medical College/Hospital, Rahim Yar Khan, PAK; 3 Orthopedic Surgery, University Hospitals Birmingham, Birmingham, GBR; 4 Trauma and Orthopedics, University Hospitals Birmingham, Birmingham, GBR; 5 Trauma and Orthopedics, Birmingham Heartland Hospital, Birmingham, GBR; 6 Trauma and Orthopedics, Hayatabad Medical Complex Peshawar, Peshawar, PAK

**Keywords:** functional outcome, mason type, mayo elbow performance score, orif, radial head fracture

## Abstract

Background: Radial head fractures (RHFs) account for a considerable injury. This study focuses on the functional results of people who had open reduction and internal fixation (ORIF).

Objective: To evaluate the functional outcomes of Mason type II and III RHFs treated with ORIF using the Mayo Elbow Performance Score (MEPS) over a 12-month postoperative period.

Methods: This retrospective study was conducted in the Orthopedic Surgery Department at Birmingham Heartlands Hospital. The research included 44 patients diagnosed with RHFs. Data regarding the patient's medical history, radiological imaging, and surgical interventions were collected from their medical records. The MEPS was utilized to assess functional outcomes at baseline and at six and 12 months postoperatively.

Results: The mean age was 42.55 ± 10.24 years. Of the total 44 patients, there were 32 (72.7%) male and 12 (27.3%) female patients. The cause of fracture was fall from height in seven (15.9%) patients, road traffic accidents in 34 (77.3%), and other in three (6.8%) patients. MEPS was measured at baseline, six months, and 12 months. There were 15 (55.6%) in type II and 10 (58.8%) in type III with excellent outcome, 10 (37%) in type II and six (35.3%) in type III with good outcome, and two (7.4%) in type II and one (5.9%) in type III with fair outcome.

Conclusion: Overall results from ORIF for RHF are favorable. After a year, the functional outcomes of patients with isolated Mason type III RHF are comparable to those of type II patients.

## Introduction

Over the past 30 years, advancements in our understanding of elbow anatomy and biomechanics have provided valuable insights into the complexities of this diarthrodial joint. The radial head plays a pivotal role in maintaining the natural and stable movement of the elbow by forming a crucial link between the bone structure and surrounding soft tissue [1]. Contrary to earlier beliefs that the radial head was a non-essential skeletal component, recent studies emphasize its importance in elbow stability, and fractures or injuries can lead to significant functional impairments if not properly treated [2]. Radial head fractures (RHFs) are relatively common, occurring at a rate of approximately 2.5-2.9 per 10,000 people annually [3]. These fractures occur across a wide demographic but are most commonly associated with high-energy trauma, such as falls on an outstretched hand or road traffic accidents [4]. Additionally, a rare but critical condition, known as floating elbow also occurs in such traumatic cases, where forearm fracture accompanies a supracondylar fracture of the humerus [5]. 

The classification of RHFs has evolved since Mason introduced the original system in 1954 [6]. The modified Mason classification, as refined by Hotchkiss, is widely used today and includes three types: type I for nondisplaced or minimally displaced fracture (<2 mm) with no mechanical block to rotation; type II for displaced fracture (>2 mm) or angulated with possible mechanical block to forearm rotation; type III for comminuted and displaced fracture with mechanical block to motion; and type IV for fractures associated with ligamentous injury or associated other fractures [7]. The Mason classification system serves as a foundation for determining the appropriate treatment plan, with more severe types (II and III) typically requiring surgical intervention [8].

In recent years, surgical options for managing RHFs have improved significantly. While non-operative treatment remains an option for some minimally displaced fractures, open reduction and internal fixation (ORIF) is now the preferred treatment for more complex fractures, particularly in younger patients [9]. ORIF allows for anatomical reduction, restoration of joint congruity, and early mobilization, which contribute to better long-term functional outcomes [10]. When ORIF is not feasible due to extensive comminution, radial head replacement or excision may be necessary [11]. The objective of this retrospective study is to evaluate the functional outcomes of Mason type II and III RHFs treated with ORIF using the Mayo Elbow Performance Score (MEPS) over a 12-month postoperative period.

## Materials and methods

This retrospective study was carried out at the Department of Orthopedic Surgery, Birmingham Heartlands Hospital. It included 44 patients who underwent surgical intervention for RHFs from January 2020 onwards. Ethical approval for the research was granted by the Institutional Ethical Review Committee of University Hospital Birmingham. Data collection involved reviewing medical records, including patient demographics, radiographic assessments, and surgical procedure details.

Inclusion criteria

Patients aged 18 to 60 years, of both genders, with Mason type II and III RHFs, confirmed via anteroposterior and lateral X-rays, treated with ORIF, and with a minimum follow-up of 12 months were included.

Exclusion criteria

Patients were excluded if they had neurological disorders, systemic comorbidities, neurological injuries, prior elbow fractures, treatment delays exceeding 10 days post-trauma, or follow-up periods shorter than 12 months.

Surgical procedure

**Figure 1:**
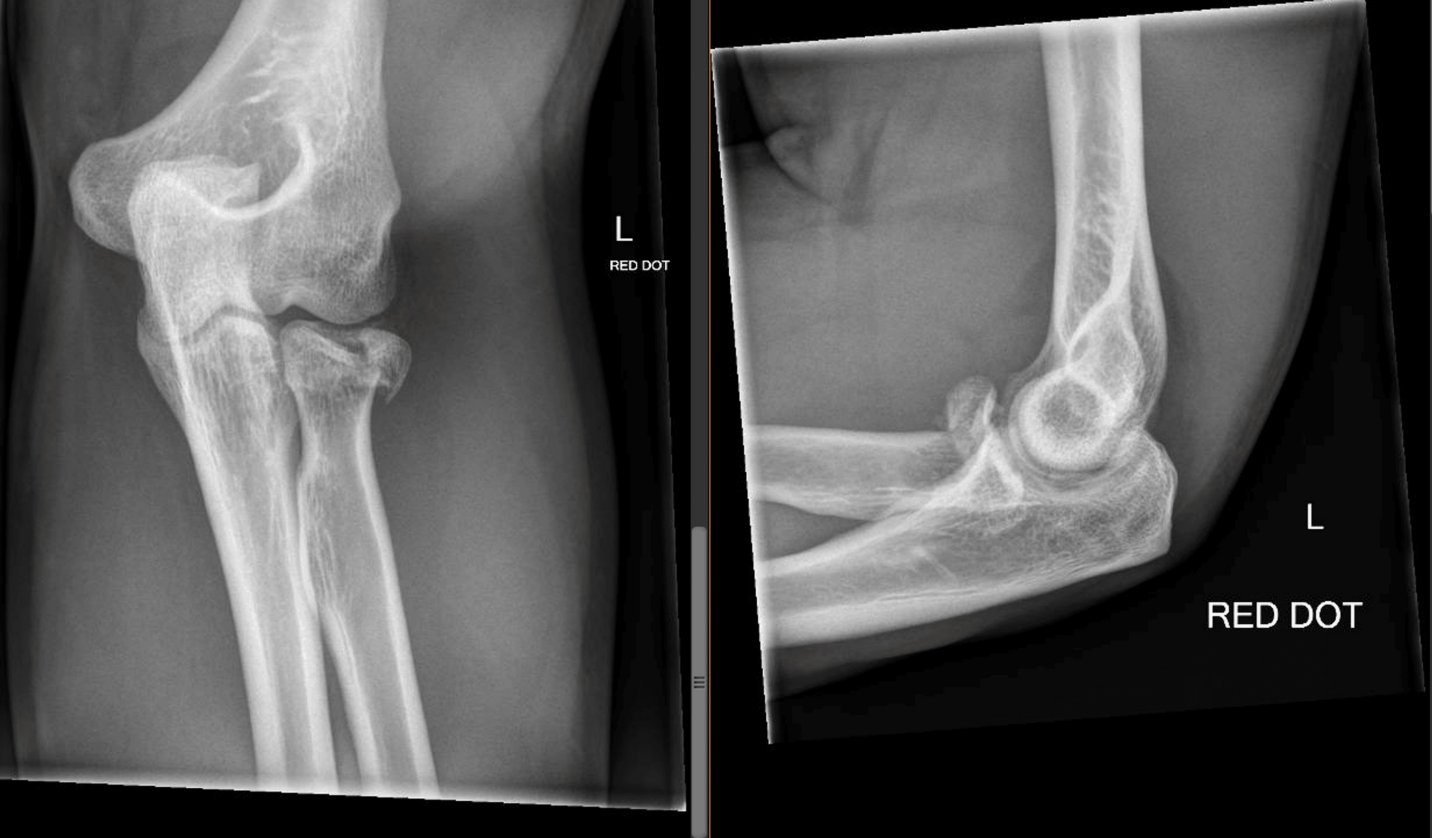
Radial head ORIF pre-op ORIF: Open reduction and internal fixation

Patients were positioned supine with forearms in pronation along with the abduction on the shoulder to keep the forearm apart from the trunk and protect the posterior interosseous nerve from any potential risk of injury. A pneumatic tourniquet was applied, and soft tissue dissection was carried out using Kocher's posterolateral approach, wherein an incision was made on the lateral aspect of the elbow, distal to the lateral epicondyle of humerus bone lowering down up to the radial head, to access the capsule of the joint between the anconeus and extensor carpi ulnaris muscle (Figure 1). 

**Figure 2:**
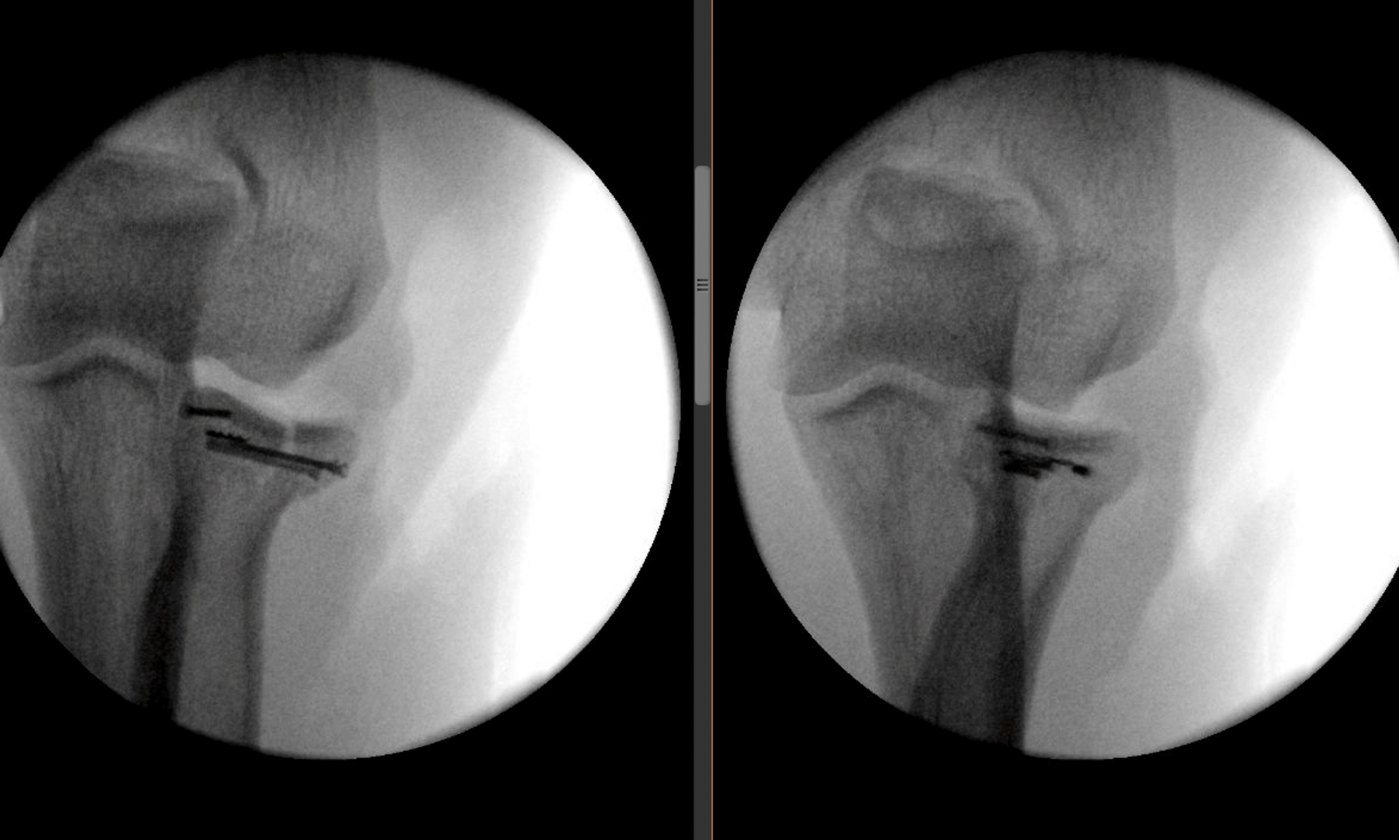
Radial head post-op

The comminuted radial head was exposed following the transection of the annular ligament and joint capsule. The safe zone for the proximal radio-ulnar joint was established for ORIF after clearing intra-articular hematoma, and osteosynthesis using mini fragment screws was performed (Figure 2). Postoperatively, patients were immobilized in a long-arm splint with neutral rotation and 90° elbow flexion.

From the second postoperative day, an elbow plaster was applied for one week, followed by passive flexion-extension and pronation-supination exercises using a hinged elbow brace. Active range of motion exercises were initiated at four weeks post-surgery. Functional outcomes were assessed at six months and one year.

Functional outcome measurement

Functional outcomes were measured using MEPS at baseline and at six and 12 months post-surgery. MEPS assesses elbow function, stability, range of motion, and pain, providing a comprehensive evaluation of recovery.

Statistical analysis

The statistical analysis was conducted using IBM SPSS Statistics version 25 (IBM Corp., Armonk, USA) to evaluate both continuous and categorical variables between patients with Mason type II and type III fractures. For continuous variables, such as age and MEPS, these were expressed as means with standard deviations to describe the central tendency and variability within each group. To compare these continuous variables between the type II and type III fracture groups, an independent t-test was applied. This test is used to determine if there is a statistically significant difference in the means of these variables between the two groups. For categorical variables, including gender, cause of injury, and the affected side (right or left), a chi-square test was employed. This test assesses whether there are significant differences in the distribution of these categorical factors between the two fracture groups. Across all analyses, a P-value of less than 0.05 was considered the threshold for statistical significance.

## Results

A total of 44 patients with closed RHFs who underwent surgical treatment at the Department of Orthopedic Surgery at Birmingham Heartlands Hospital over the last four years were included in the study. The mean age for the type II group was an average of 44 years (±10.25) and for the type III group was 40.24 years (±10.05). A t-test (T-value = 1.207, P-value = 0.239) and F-test (F-value = 1.460) indicated no significant difference in mean age or variability between the groups. Regarding gender, 62.5% of type II and 58.3% of type III patients were male. A chi-square test (Chi-square = 0.389, P-value = 0.533) showed no significant difference in gender distribution. The most common cause of injury was road traffic accidents (61.8% in type II, 38.2% in type III). A chi-square test (Chi-square = 2.897, P-value = 0.234) again showed no significant difference between the groups. Lastly, the side of injury (right or left) was compared, with 66.7% of type II and 33.3% of type III patients having right-side injuries. A chi-square test (Chi-square = 0.742, P-value = 0.389) found no significant difference in side distribution (Table 1).

**Table 1:** Patients characteristics, type, and mechanism of injury RTA: Road traffic accident

Parameters	Type II (n=27)	Type III (n=17)	P-value	Statistical Test
Age (years)	44 ± 10.25	40.24 ± 10.05	0.239	T-value = 1.207, F-value = 1.460
Gender			0.533	Chi-square = 0.389
Male	20 (62.5%)	12 (37.5%)		
Female	7 (58.3%)	5 (41.7%)		
Cause			0.234	Chi-square = 2.897
Fall	3 (42.9%)	4 (57.1%)		
RTA	21 (61.8%)	13 (38.2%)		
Other	3 (100%)	0 (0%)		
Side			0.389	Chi-square = 0.742
Right	12 (66.7%)	6 (33.3%)		
Left	15 (57.7%)	11 (42.3%)		

MEPS were measured at baseline, six months, and 12 months to compare outcomes between patients with type II and type III fractures. At baseline, the average MEPS was 44.96 for type II and 46.24 for type III, with a P-value of 0.073, indicating no significant difference between the groups at this early stage. At the six-month mark, the scores for both groups were nearly identical, with type II at 64.52 and type III at 64.59, and a P-value of 0.970, again showing no statistically significant difference. By 12 months, both groups showed substantial improvement, with MEPS rising to 86.48 for type II and 88.35 for type III. However, the P-value of 0.278 indicated that this difference was not statistically significant (Table 2).

**Table 2:** Mean MEPS at baseline, six months, and 12 months MEPS: Mayo Elbow Performance Score

Time Point	Type II (Mean ± SD)	Type III (Mean ± SD)	P-value	T-value	F-value
Baseline	44.96 ± 2.50	46.24 ± 1.72	0.073	1.837	3.374
Six months	64.52 ± 7.03	64.59 ± 5.19	0.970	0.038	0.001
12 months	86.48 ± 6.05	88.35 ± 5.12	0.278	1.104	1.224

A score between 90 to 100 was considered excellent, 75-89 good, and 60-74 fair. In the type II group, 15 (55.6%) had an excellent outcome, 10 (37%) had a good outcome, and two (7.4%) had a fair outcome. In the type III group, 10 (58.8%) had an excellent outcome, six (35.3%) had a good outcome, and one (5.9%) had a fair outcome. There was no significant difference between the two groups in terms of outcomes (P-value = 0.969, Chi-square = 0.063) (Table 3).

**Table 3:** Final functional outcome in type II and type III patients

Parameters	Type II (n=27)	Type III (n=17)	Total (n=44)	P-value	Chi-square
Excellent	15 (55.6%)	10 (58.8%)	25 (56.8%)		
Good	10 (37.0%)	6 (35.3%)	16 (36.4%)		
Fair	2 (7.4%)	1 (5.9%)	3 (6.8%)		
Total	27 (100.0%)	17 (100.0%)	44 (100.0%)	0.969	0.063

## Discussion

RHFs make up 1.5-4% of all fractures in adults. While Mason type I and some type II fractures can often be treated without surgery, more serious cases like Mason type II, III, and IV usually need surgery. The choice of the method to operate either ORIF, arthroplasty, or radial head removal depends on the number, size, angulation, and displacement of the fracture fragments, with each method leading to different results [8]. Moreover, this depends upon the type of RHF, where ORIF is given preference to lower the risk of capitellar erosion and alteration in radioulnar biomechanics, while in the conservative approach, it is crucial to look for the presence of a mechanical block that necessitates going for open reduction, especially in young patients [12]. 

This retrospective study looks at the results of Mason type II and III RHFs treated with ORIF. This includes the realignment of the fractured bony fragments in anatomical order through manual manipulation using clamps or reduction forceps, then mini fragment screws were used for the fixation. The average age of the patients was 42.55 years. Out of 44 patients, 32 (72.7%) were men and 12 (27.3%) were women. The mechanisms for fractures were falls from height in seven patients (15.9%), road traffic accidents in 34 patients (77.3%), and other causes in three patients (6.8%). These results match earlier studies that found similar causes for RHFs [13,14].

Both Mason type II and III fractures demonstrated comparable functional outcomes following surgical management, with no statistically significant differences between the groups. These findings align with previous studies on this subject [15,16].

A retrospective study compared the non-operative and operative treatments for Mason type II fractures with 2-5 mm displacement, showing no significant clinical difference, with mean Quick Disabilities of the Arm, Shoulder, and Hand (QuickDASH) scores of seven and six, respectively (P = 0.90) [17]. Zwingmann et al. [18], in a meta-analysis of 33 studies including 302 patients, reported that ORIF had a 92% success rate, although the difference compared to other treatment methods was not statistically significant (P = 0.266).

In a systematic review by Zhao et al., a total of 271 patients from four studies were included, with 142 patients receiving surgical treatment and 129 receiving non-surgical treatment. The findings indicated no statistically significant differences between the two treatment groups in terms of the Disabilities of the Arm, Shoulder, and Hand (DASH) score, Oxford Elbow Score (OES), MEPS, elbow flexion and extension impairments, or elbow pain. However, non-surgical treatment was associated with certain advantages. Specifically, it led to greater elbow pronation (OR = -3.10; 95% CI = -4.96, -1.25; P = 0.55; I² = 0%) and a lower complication rate (OR = 5.54; 95% CI = 1.79, 17.14; P = 0.42; I² = 0%) compared to surgical treatment, highlighting the importance of conservative treatment in Mason type II RHFs [19].

Further support for ORIF is provided by a large single-center case series, which demonstrated that most patients had satisfactory or excellent results at 30 months of follow-up [20]. Another study reported a 22% loss to follow-up at 24 months, highlighting the challenge of long-term data collection in these patient populations [21]. Additionally, a separate investigation involving 45 patients treated with ORIF showed predominantly satisfactory and excellent outcomes after an average follow-up period of 17 months [22,23].

Limitations

Our study has several limitations. As a retrospective study, we were unable to follow patients at multiple intervals to assess the progression of functional scores over time. The relatively small sample size may limit the generalizability of our findings. Additionally, the lack of a control group prevents a direct comparison between ORIF and other treatment options like radial head arthroplasty or excision, especially for type III RHFs. Lastly, the follow-up period was limited to one year, which may not capture long-term outcomes or complications such as implant failure or post-traumatic arthritis.

Despite these limitations, our study has several strengths. The consistent use of ORIF across all cases allows for a focused evaluation of its efficacy in treating Mason type II and III fractures. Furthermore, the standardized rehabilitation protocol ensures that differences in functional outcomes are attributable to the surgical intervention rather than postoperative care variations. These factors contribute to the robustness of our findings and support the conclusion that ORIF is an effective treatment modality for these types of fractures.

## Conclusions

The study concludes that overall results from ORIF for RHFs are favorable. After a year, the functional outcomes of patients with isolated Mason type III RHF were comparable to those of type II patients, demonstrating that even more complex fractures can achieve similar levels of recovery with appropriate surgical intervention. With high rates of good to exceptional functional results, ORIF was shown in most patients to restore joint stability, increase range of motion, and lower discomfort. These findings confirm the main treatment option for displaced fractures by underlining the efficiency of ORIF in controlling both Mason type II and III RHFs. Long-term research is also essential to monitor for any late problems such as arthritis or implant failure and to assess the lifetime quality of these results.

ORIF, being an effective reduction approach for type II and III fractures according to the Mason classification of RHFs, ensured optimal outcomes in almost 90% of the cases in a duration of 12 months on MEPS, and besides the potential complexities of these fracture types, it helped restore the range of motion, pain relief, stability, and joint integrity. Moreover, this study, despite its retrospective design, provides reinforcement to the existing literature in support of ORIF as an optimal surgical option for both of these fracture types along with positive long-term outcomes. 
